# Disentangling the Association between ADHD and Alcohol Use Disorder in Individuals Suffering from Bipolar Disorder: A Systematic Review and Meta-Analysis

**DOI:** 10.3390/brainsci12010038

**Published:** 2021-12-28

**Authors:** Francesco Bartoli, Tommaso Callovini, Angela Calabrese, Riccardo M. Cioni, Ilaria Riboldi, Cristina Crocamo, Giuseppe Carrà

**Affiliations:** 1 Department of Medicine and Surgery, University of Milano Bicocca, Via Cadore 48, 20900 Monza, Italy; francesco.bartoli@unimib.it (F.B.); a.calabrese16@campus.unimib.it (A.C.); r.cioni1@campus.unimib.it (R.M.C.); i.riboldi1@campus.unimib.it (I.R.); cristina.crocamo@unimib.it (C.C.); giuseppe.carra@unimib.it (G.C.); 2 Division of Psychiatry, University College London, Maple House 149, London W1T 7BN, UK

**Keywords:** ADHD, bipolar disorder, alcohol use disorder, dual diagnosis, mood disorders

## Abstract

Attention Deficit-Hyperactivity disorder (ADHD) may influence rates of Alcohol Use Disorder (AUD) among individuals suffering from Bipolar Disorder (BD). The aim of this systematic review and meta-analysis was to estimate the strength and consistency of the potential association between ADHD and AUD in BD. We searched main electronic databases for studies indexed up to November 2020. We included observational studies investigating the association between ADHD and AUD among individuals with BD. The association between ADHD and AUD was estimated using odds ratios (ORs) with 95% Confidence Intervals (CIs). Eleven studies, involving 2734 individuals with BD (516 with ADHD), were included in the meta-analysis. Individuals with both BD and ADHD had higher rates of AUD as compared with subjects with BD only (34.0% vs. 18.3%). The estimated OR of AUD for ADHD was 2.50 (95% CI: 1.91 to 3.27; I^2^ = 13.0%). Study-level characteristics did not influence the effect size. No risk of publication bias was estimated. Despite some limitations, this meta-analysis estimated an association between ADHD and AUD among individuals suffering from BD. At least a portion of the high rates of AUD in BD may, thereby, be related to comorbid ADHD. Longitudinal studies are needed to clarify the nature of this relationship.

## 1. Introduction

Bipolar disorder (BD) is a severe mental illness affecting about 2–3% of the population worldwide [1]. Individuals with BD are likely to have clinical comorbidities associated with a more severe course of illness [2]. Among them, alcohol use disorder (AUD) is one of the most frequent, involving about one quarter of individuals suffering from BD [3]. The comorbidity between AUD and BD leads to worse clinical outcomes, possibly characterized by poor response to treatment [4], mood instability and greater symptom severity [5], increased suicidality [6], heightened risk of hospitalization [7,8], and cognitive dysfunctions [9]. Among different clinical correlates perhaps explaining this comorbidity, a role for ADHD, which is, in turn, a frequent co-occurring condition in BD [10,11], can be hypothesized. Recent systematic reviews and meta-analyses [10,11] estimated that ADHD might occur in nearly one out of six adults with BD, possibly anticipating its onset by about four years [11]. It is well known that ADHD itself might be correlated with AUD [12,13]. Previous longitudinal studies have shown that ADHD might have a significant impact on alcohol-related behaviors [13,14]. The high levels of impulsivity and sensation seeking that has been described among individuals with ADHD may significantly increase the risk of AUD [15]. In addition, prospective data highlighted that both the attenuation of negative affect and the enhancement of positive mood or wellbeing, might represent possible clinical mediators of the association between ADHD and AUD [14]. Moreover, observational studies have shown higher rates of problematic alcohol use in adults suffering from ADHD, with genetic links primarily underlying this association [16]. Despite the clinical and epidemiological interconnections between these disorders, the possible impact of ADHD on AUD rates in BD has not been systematically analyzed so far. Consequently, we conducted a systematic review and meta-analysis of observational studies to estimate the strength and consistency of the association between ADHD and AUD among individuals suffering from BD.

## 2. Materials and Methods

### 2.1. Search Strategy and Inclusion Criteria

The current systematic review and meta-analysis was conducted according to the Preferred Reporting Items for Systematic Reviews and Meta-Analyses (PRISMA) Statement (Supporting Information) [17]. We searched Medline, Embase, and PsycInfo electronic databases (via Ovid) for studies published up to November 2020. The following search phrase was used: *((Bipolar or Mania) and ADHD)* as a multiple purpose search in title, abstract, heading words, and keywords. No language restriction was applied. An additional, post hoc non-systematic search of studies indexed in Google Scholar was made in order to check if additional studies were retrievable. We included observational studies analyzing rates of current or lifetime AUD in adults with BD with and without ADHD, respectively. We excluded studies involving >10% of subjects aged <18 years old. We also excluded studies with incomplete data, such as conference abstracts, dissertations, and grey literature, not undergoing peer review. If data from the same sample were published in multiple works, we retained the article reporting more comprehensive information, to avoid data duplication [18]. Three authors (TC, AC, and RMC) independently completed the preliminary screening based on titles and abstracts, retrieved full texts for the final assessment of study eligibility, and recorded reasons for article exclusion. Possible disagreements in the study assessment were resolved by discussion with a fourth author (FB).

### 2.2. Data Extraction

We extracted key information from the eligible studies, i.e., year of publication, country, setting, methods to assess ADHD and AUD, timeframe (adult or childhood) of comorbid ADHD, main characteristics of index/control groups, and main findings. Three authors (TC, AC, and RMC) independently extracted data for a blind check of accuracy. Authors of studies with unclear or partial data were contacted by e-mail for additional information in order to reduce the risk of selective reporting bias and to also include unpublished findings.

### 2.3. Quality Assessment

We ascertained the possible occurrence of selection bias [19], checking if compared groups (BD with vs. BD without ADHD) were similar in terms of main characteristics, i.e., age and gender, considering acceptable a difference of maximum 3 years in mean age and 5% in male gender proportion, respectively. In addition, we carried out an assessment of potential sources of information and misclassification bias [20,21]. First, we checked whether studies used adequate instruments to assess ADHD, such as the Diagnostic Interview for ADHD in Adults (DIVA) [22] or the Wender Utah Rating Scale (WURS) [23] as well as other appropriate diagnostic interviews [24]. Second, we evaluated whether studies used appropriate diagnostic interviews to assess AUD, such as the Structured Clinical Interview for DSM (SCID) [25] or the MINI-International Neuropsychiatric Interview (MINI) [26], instead of diagnoses based on non-structured, clinical evaluations or clinical chart/databases review. Third, since a diagnosis of ADHD during manic or depressive phases is not recommended [27], we checked if individuals were tested in euthymia or during mood episode remission.

### 2.4. Data Analysis

We estimated the prevalence rates (arcsine transformed proportions), with 95% Confidence Intervals (CIs), of AUD in BD individuals with and without ADHD, respectively. The association between ADHD and AUD was estimated using the odds ratios (ORs) with 95% CIs. Study weights were obtained using random-effects models for meta-analyses. Heterogeneity between studies was evaluated using standard cut-offs for the I^2^ statistic, with values of 25%, 50%, or 75% defining different levels of inconsistency (low, moderate, or high). Publication bias was assessed using the Egger’s test with relevant *p*-value. Meta-regression analyses of study-level data were carried out, using Monte Carlo permutation test to assess whether selected characteristics might influence the effect estimates. These included the year of publication (before vs. in/after 2014); country/geographical area; setting (outpatients vs. other); sample size (as a continuous variable); mean age (as a continuous variable); proportion of women (as a continuous variable); proportion of BD type 1 (as a continuous variable); number of quality criteria met (≥3 vs. <3). Additional intra-group analyses were made to test if comorbid current and past ADHD differently influenced the association between BD and AUD. All *p*-values were two-sided and considered significant when *p* < 0.05. Analyses were performed in Stata statistical software package 15 [28].

## 3. Results

### 3.1. Study Selection

Our search generated 4645 records from selected electronic databases (1048 from Medline, 2270 from Embase, and 1327 from PsycInfo). After removing duplicates, 2926 articles were identified. The preliminary screening of titles and abstracts identified 32 potentially eligible articles, including also an additional study retrieved in Google Scholar. According to the final eligibility assessment of full texts, 11 studies were included [29,30,31,32,33,34,35,36,37,38,39], while 21 studies did not meet inclusion criteria. We benefited also from additional, unpublished data provided by the authors of two studies [29,31].

**Figure 1:**
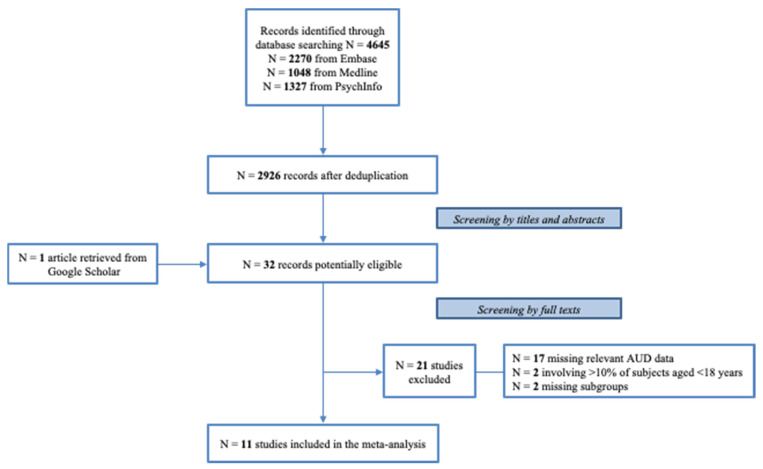
Flow chart of the included and excluded studies.

The flowchart with screening details and reasons for exclusion is shown in Figure 1.

### 3.2. Study Characteristics

Studies included in this meta-analysis were published between 2005 [33] and 2019 [37]. Three studies were conducted in Turkey [30,31,38], two in India [29,34], two in North America [32,33], and the others in different European countries, namely Italy [36,37], Spain [39], and Switzerland [35]. Sample sizes ranged from 90 [30] to 919 individuals [33]. Most of the studies selected outpatients only [29,30,31,33,35,36,37,38,39], whereas two studies included both inpatients and outpatients [32,34]. Four studies assessed individuals for current ADHD [31,32,34,35], whereas seven studies for lifetime ADHD [29,30,33,36,37,38,39] with four [29,30,38,39] providing separate data for adult and childhood ADHD. The characteristics of the included studies are reported in Table 1. In terms of quality assessment, in only four [29,32,33,38] and three studies [35,36,38], respectively, there was a sufficient comparability of age and gender between individuals with and those without ADHD. All studies used valid instruments for diagnosis of ADHD, whereas five of them used unclear methods to assess AUD [29,30,31,34,37]. ADHD was assessed during the euthymic phase in most studies, except for three [33,35,36], whereas no information on mood phase at the time of assessment was reported in two studies [32,37]. The summary of quality assessment is reported in the Table S1a,b.

### 3.3. Association between Comorbid ADHD and Alcohol Use Disorder

**Figure 2:**
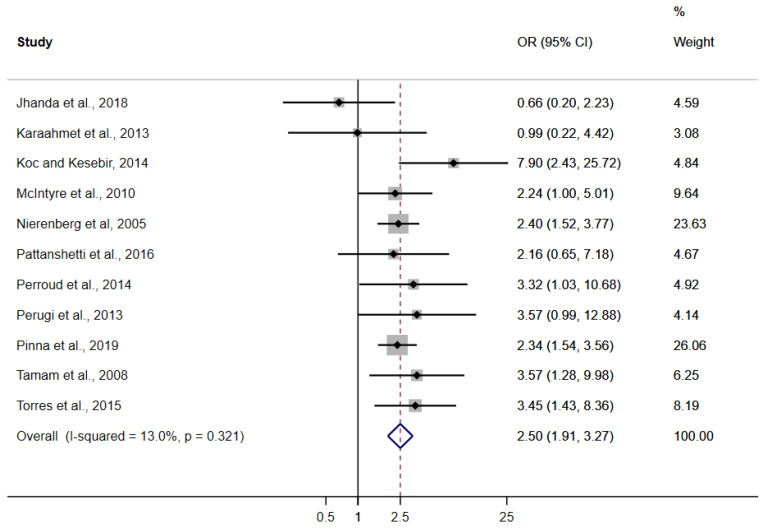
Association between ADHD and Alcohol Use Disorder in Bipolar Disorder.

The random-effects meta-analysis, including 11 studies [29,30,31,32,33,34,35,36,37,38,39] based on 2734 individuals with BD, showed that individuals with both BD and ADHD (*n* = 516) had higher rates of AUD as compared with subjects with BD only (*n* = 2218) (arcsine transformed proportions: 34.0% [95% CI: 21.7 to 47.4%] vs. 18.3% [95% CI: 10.1 to 28.4%]). The estimated OR was 2.50 (95% CI: 1.91 to 3.27; I^2^ = 13.0%) (Figure 2). Meta-regression analyses showed that study-level characteristics did not influence the effect size (Table 2). The Egger’s test did not estimate any risk of publication bias (*p* = 0.91). Finally, intragroup analysis, based on four studies [29,30,38,39] and 163 individuals with both BD and ADHD, showed no differences in AUD rates between current (adult) and past (childhood) ADHD (OR: 1.86; 95% CI: 0.71 to 4.89; I^2^ = 0%).

## 4. Discussion

To our knowledge, this is the first systematic review and meta-analysis aimed at clarifying the strength and consistency of the relationship between ADHD and AUD among individuals suffering from BD. Based on data from 11 observational studies, accounting for 2734 subjects with BD (516 with and 2218 without ADHD, respectively), we estimated an association between AUD and ADHD. Individuals with ADHD - representing about one out of six individuals with BD, consistently with available epidemiological data [10,11] - were more than twice as likely to report AUD as compared to those with BD only. The low heterogeneity across studies, the precision of the effect size, as well as the lack of study-level characteristics influencing the magnitude of the effect, corroborated the robustness of our findings. In addition, relevant statistics showed a low probability of publication bias. Our results were substantiated by additional analyses which showed that the likelihood of comorbid AUD was not different comparing BD individuals with adult ADHD and those with childhood ADHD, even though data were based on few studies and a small sample size.

Additional research is needed to clarify which clinical factors might influence or mediate this relationship. For example, it has been hypothesized that high levels of impulsivity in BD might represent the core symptom shared with ADHD and a driving factor for AUD [40,41]. In particular, it has been reported that attention deficits occurring in both mania and ADHD might induce the hyperactive, impulsive and sensation-seeking behaviours as autoregulatory patterns [42]. On the other hand, underlying neurobiological substrates and genetic correlates, which might be shared between BD, ADHD, and AUD, have been hypothesized. For example, there is some evidence that dysfunction of dopamine in the brain reward circuitry might link co-occurring ADHD, mood and substance use disorders [43]. Consistently, it has been proposed that vulnerability to AUD in ADHD might be due to genetic mutations in the dopamine receptors as well as abnormalities in the prefrontal brain area designed for planning and reasoning [44]. In addition, functional imaging studies have shown that there may be abnormalities in specific brain regions, in particular the frontal-subcortical circuits, in individuals with ADHD and in those with addictive behaviours [45], even though the link with BD seems less clear.

In terms of clinical implications, our findings highlight the importance of a careful assessment of AUD among individuals with comorbid ADHD and BD in routine clinical practice. The proposed hierarchical therapeutic approach [46,47] based, first, on mood stabilization and only afterwards on ADHD symptom management, using, e.g., stimulants or atomoxetine [48], might be further complicated by the presence of AUD. Indeed, additional caution has been suggested in the use of psychostimulants for the treatment of ADHD symptoms among individuals with addictive behaviors [49], also considering their potential pharmacodynamic interactions with ethanol [48]. Furthermore, findings from clinical studies have shown that treatment with atomoxetine, though relatively safe [50], may have inconsistent effects for alcohol-related behaviors in people with ADHD [51], even if the related improvement of ADHD symptoms might correlate with a reduction of alcohol craving [52]. As a whole, it is likely that the clinical management of BD with co-occurring ADHD and AUD would require more intensive monitoring and treatment [6]. The screening of these comorbidities in BD represents a key clinical challenge considering that people with co-occurring mental disorders and addictive behaviors are generally less likely to be identified and to receive appropriate and effective treatments [6], possibly based on integrated approaches [53]. Moreover, since the validity of comorbid ADHD diagnosis in BD is not well-established [54,55], its assessment would require careful attention to phenomenology, childhood history, and lifetime course of symptoms [56]. The epidemiological burden of the comorbidity between BD and ADHD, as well as the potential impact of AUD on clinical outcomes of both disorders [4], calls for further clinical research in order to better define evidence-based treatment algorithms.

Despite that the magnitude and strength of the estimated association seem to highlight a significant burden of ADHD for comorbid AUD in BD, the findings of this systematic review and meta-analysis should be interpreted with caution, and some limitations and methodological issues should be taken into account. First, as our meta-analysis is based only on cross-sectional data, no causal inference can be made for the association between ADHD and AUD in BD. Prospective data are needed to define the potential role of ADHD as a clinical factor contributing to the onset of an AUD among individuals with BD. Second, our work provided a categorical evaluation of the association between AUD and ADHD in individuals suffering from BD, without any additional information on frequency and severity of patterns of alcohol use. This issue is particularly important considering that ADHD symptoms might predict the severity of alcohol-related problems [57] or a polysubstance abuse pattern [58]. Moreover, an increase in alcohol consumption might be associated with specific features of ADHD, such as attentional bias [59].

Finally, some methodological issues across studies included in this meta-analysis should be considered, in terms of potential selection and information bias. In particular, standardized interviews for AUD evaluation were used in a portion of studies, and age and gender between ADHD and non-ADHD samples were comparable in few studies only. Moreover, some studies assessed ADHD during any mood phase, making the diagnosis of ADHD more complex because of overlapping symptoms, such as distractibility, talkativeness, irritability, and emotional lability [27]. Nonetheless, it should be noted that our findings did not show any moderating role by relevant quality items on the estimated sizes of the effects.

As a whole, despite some limitations, this meta-analysis could estimate an association between ADHD and AUD among individuals suffering from BD. Although ADHD may explain only a portion of AUD rates in BD, we could uncover an important burden of this clinical relationship in BD. Considering the possible interactions between these disorders, additional research is needed to define effective approaches and treatments for BD individuals suffering from these comorbid conditions.

## Figures and Tables

**Table 1:** Characteristics of studies included in the meta-analysis.

Study [Reference]	Country	Setting	Sample Size N	Age Mean (SD), yrs	Females *n*/N (%)	BD-I *n*/N (%)	ADHD *n*/N (%)	AUD *n*/N (%)
Jhanda et al., 2018 [29]	India	Outpatients	99	29.0 (5.7)	27/99 (27.3)	-	30/99 (30.3)	17/99 (17.2)
Karaahmet et al., 2013 [30]	Turkey	Outpatients	90	36.5 (10.8)	42/90 (46.7)	-	34/90 (37.8)	8/90 (8.9)
Koc and Kesebir, 2014 [31]	Turkey	Outpatients	114 ^†^	42.8 (12.5)	70/114 (61.4)	-	30/114 (26.3)	15/114 (13.2)
McIntyre et al., 2010 [32]	Canada and US	Inpatients/ outpatients	176	38.7 (12.6)	112/169 (66.3) ^‡^	119/175 (68.0) ^§^	31/176 (17.6)	85/176 (48.3)
Nierenberg et al., 2005 [33]	US	Outpatients	919	40.6 (12.8)	536/917 (58.5) ^¶^	636/919 (69.2)	87/919 (9.5)	381/919 (41.5)
Pattanshetti et al., 2016 [34]	India	Inpatients/ outpatients	100	37.4 (11.0)	36/100 (36.0)	-	15/100 (15.0)	21/100 (21.0)
Perroud et al., 2014 [35]	Switzerland	Outpatients	138	42.0 (11.9)	76/138 (55.1)	47/138 (34.1)	27/124 (21.8) ^∫^	20/131 (15.3) ^√^
Perugi et al., 2013 [36]	Italy	Outpatients	96	42.3 (13.5)	39/96 (40.6)	64/96 (66.7)	19/96 (19.8)	12/96 (12.5)
Pinna et al., 2019 [37]	Italy	Outpatients	703	46.0 (15.6)	386/703 (54.9)	368/703 (52.3)	173/703 (24.6)	117/703 (16.6)
Tamam et al., 2008 [38]	Turkey	Outpatients	159	33.7 (10.3)	79/159 (49.7)	146/159 (91.8)	43/159 (27.0)	17/159 (10.7)
Torres et al., 2015 [39]	Spain	Outpatients	163	42.7 (12.5)	88/163 (54.0)	123/163 (75.5)	29/163 (17.8)	78/161 (48.4) ^∅^

ADHD = Attention Deficit Hyperactivity Disorder; AUD = Alcohol Use Disorder; BD-I = Bipolar I Disorder; SD = standard deviation; US = United States. ^†^ Individuals with ADHD sub-dimensions, i.e., attention deficit and hyperactivity, were excluded from analyses; ^‡^ Information on gender not available in seven individuals; ^§^ Information on presence/absence of BD-I not available in one individual; ^¶^ Information on gender not available in two individuals; ^∫^ Information on presence/absence of ADHD not available in 14 individuals; ^√^ Information on presence/absence of AUD not available in seven individuals; ^∅^ Information on presence/absence of AUD not available in two individuals.

**Table 2:** Influence on effect size of study-level characteristics: meta-regression analyses.

Study Characteristics	k	Coefficient	Standard Error	*p*-Value
Year of publication [before (k = 5) vs. in/after (k = 6) 2014]	11	0.055	0.320	0.843
Geographical area [vs. India (k = 2)]	11			
Europe	4	0.807	0.489	0.191
Turkey	3	1.085	0.576	0.105
North America	2	0.672	0.501	0.309
Setting [inpatients/outpatients (k = 2) vs. outpatients (k = 9)]	11	0.148	0.433	0.600
Sample size *	11	0.000	0.000	0.727
Mean age *	11	0.042	0.031	0.110
Proportion of BD-I *	7	0.005	0.010	0.445
Proportion of women *	11	0.022	0.015	0.073
Quality [<3 (k = 5) vs. ≥3 (k = 6) quality criteria]	11	−0.026	0.332	0.939

BD-I = Bipolar I Disorder; k = number of studies; * tested as a continuous variable.

## Data Availability

The corresponding author can provide raw data used for this meta-analysis on request.

## Supplementary Materials

The following are available online at https://www.mdpi.com/article/10.3390/brainsci12010038/s1, Table S1: Quality assessment of included studies.
