# Supplementary material for: Disentangling the Association between ADHD and Alcohol Use Disorder in Individuals Suffering from Bipolar Disorder: A Systematic Review and Meta-Analysis
Source: Brain Sci. 2021 Dec 28;12(1):38. doi: 10.3390/brainsci12010038 (PMC8773515; doi:10.3390/brainsci12010038)
Supplement: Supplementary file 1 [file brainsci-12-00038-s001.zip › brainsci-1516606-supplementary.pdf]

**Table S1. Quality assessment of included studies**

**a)**

| **Study** | **Age comparability** [a] | **Gender comparability** [b] | **ADHD diagnosis** [c] | **AUD diagnosis**[d] | **Mood phase**[e] |
|---|---|---|---|---|---|
| Jhanda et al., 2018 | + | - | + | ? | + |
| Karaahmet et al., 2013 | - | - | + | ? | + |
| Koc and Kesebir, 2014 | - | - | + | ? | + |
| McIntyre et al., 2010 | + | - | + | + | ? |
| Nierenberg et al., 2005 | + | - | + | + | - |
| Pattanshetti et al., 2016 | ? | ? | + | - | + |
| Perroud et al., 2014 | - | + | + | + | - |
| Perugi et al., 2013 | - | + | + | + | - |
| Pinna et al., 2019 | - | - | + | - | ? |
| Tamam et al., 2008 | + | + | + | + | + |
| Torres et al., 2015 | - | - | + | + | + |

ADHD = Attention Deficit Hyperactivity Disorder; AUD = Alcohol Use Disorder; [a] difference in mean age ≤ 3 years = +; difference in mean age > 3 years = –; Unclear = ?; [b] difference in gender proportion ≤ 5% = +; difference in gender proportion > 5% = –; unclear = ? [c] adequate instruments to assess ADHD = +; inadequate instruments to assess ADHD = –; unclear = ?; [d] appropriate diagnostic interviews to assess AUD = +; diagnoses of AUD based on non-structured, clinical evaluations or clinical chart / databases review = –; unclear = ?; [e] ADHD assessment in euthymia or during mood episode remission = +; ADHD assessment without mood state restriction = –; unclear = ?.

**b)**

| Study | Age comparability *(Mean age in the two groups)* | Gender comparability *(% of males in the two groups)* | ADHD diagnosis | AUD diagnosis | Mood phase |
|---|---|---|---|---|---|
| Jhanda et al., 2018 | BD + ADHD = 27.4<br>BD = 29.8 | BD + ADHD = 83.3 %<br>BD = 68.1 % | MINI DSM-IV | Unclear | Euthymia / in remission |
| Karaahmet et al., 2013 | BD + ADHD =34.3<br>BD = 37.8 | BD + ADHD = 67.6 %<br>BD = 44.6 % | SCID-I;<br>Turgay's Adult ADD/ADHD Scale;<br>WURS | Unclear | Euthymia / in remission |
| Koc and Kesebir, 2014 | BD + ADHD = 37.3<br>BD = 44.7 | BD + ADHD = 46.7 %<br>BD = 35.7 % | Adult Attention Deficit and Hyperactivity Rating Scale | Unclear | Euthymia / in remission |
| McIntyre et al., 2010 | BD + ADHD = 36.7<br>BD = 39.1 | BD + ADHD = 38.7 %<br>BD = 32.6 % | ASRS-1.1;<br>MINI plus 5.0.0;<br>WURS-25 | MINI plus 5.0.0 | Unclear |
| Nierenberg et al., 2005 | BD + ADHD = 37.8<br>BD = 40.9 | BD + ADHD = 64.4 %<br>BD = 39.2 % | MINI 4.4 | MINI 4.4 | No mood restrictions |
| Pattanshetti et al., 2016 | N/R | N/R | ASRS-1.1;<br>ASRS-1.1 Symptom Checklist | diagnoses based on non-structured, clinical evaluations or clinical chart / databases re-view. | Euthymia / in remission |
| Perroud et al., 2014 | BD + ADHD = 37.2<br>BD = 43.2 | BD + ADHD = 44.4 %<br>BD = 41.2 % | ASRS-1.1 Symptom Checklist;<br>DIVA | MINI | No mood restrictions |
| Perugi et al., 2013 | BD + ADHD = 38.5<br>BD = 43.2 | BD + ADHD = 63.2 %<br>BD = 58.4 % | ASRS-1.1 | DSM-IV-TR diagnostic criteria | No mood restrictions |
| Pinna et al., 2019 | BD + ADHD = 39.9<br>BD = 47.9 | BD + ADHD = 65.9 %<br>BD = 38.3 % | ASRS-1.1;<br>DSM-IV-TR criteria | diagnoses based on non-structured, clinical evaluations or clinical chart / databases re-view. | Unclear |
| Tamam et al., 2008 | BD + ADHD = 33.8<br>BD = 33.8 | BD + ADHD = 51.2 %<br>BD = 50 % | K-SADS-PL;<br>SCID;<br>WURS-25 | SCID-I | Euthymia / in remission |
| Torres et al., 2015 | BD + ADHD = 39<br>BD = 43.6 | BD + ADHD = 58.6 %<br>BD = 42.5 % | ASRS-1.1;<br>CAADID;<br>WURS | SCID-I | Euthymia / in remission |

ADHD = Attention Deficit Hyperactivity Disorder; AUD = Alcohol Use Disorder; ASRS-1.1 = Adult Attention Deficit and Hyperactivity Self-Report Scale, version 1.1; CAADID = Conners' Adult ADHD Diagnostic Interview for DSM-IV; DIVA = Diagnostic Interview for ADHD in adults; DSM-IV = Diagnostic and Statistical Manual of Mental Disorders, fourth edition; DSM-IV-TR = Diagnostic and Statistical Manual of Mental Disorders, fourth edition, Text Revision; K-SADS-PL = Kiddie Schedule of Affective Disorders and Schizophrenia - Present/Lifetime; MINI = Mini International Neuropsychiatric Interview; MINI 4.4 = Mini International Neuropsychiatric Interview plus, version 4.4; MINI plus 5.0.0 = Mini International Neuropsychiatric Interview plus, version 5.0.0; N/R = Not Rated; SCID = Structured Clinical Interview; SCID-I = Structured Clinical Interview Axis I Disorders; Turgay's Adult ADD/ADHD Scale = Turgay's Turkish version of Adult ADD/ADHD DSM IV-Based Diagnostic Screening and Rating Scale; WURS = Wender Utah Rating Scale; WURS-25 = Wender Utah Rating Scale - 25-item version.
